# Claw Hand in Leprosy

**DOI:** 10.4269/ajtmh.20-0701

**Published:** 2020-10

**Authors:** Juan Carlos Cataño, Marcela Tabares

**Affiliations:** 1 Infectious Diseases Section, Internal Medicine Department, University of Antioquia School of Medicine, Medellín, Colombia;; 2 Infectious Diseases Section, Clinica Las Vegas, Medellín, Colombia

**Figure 1.**
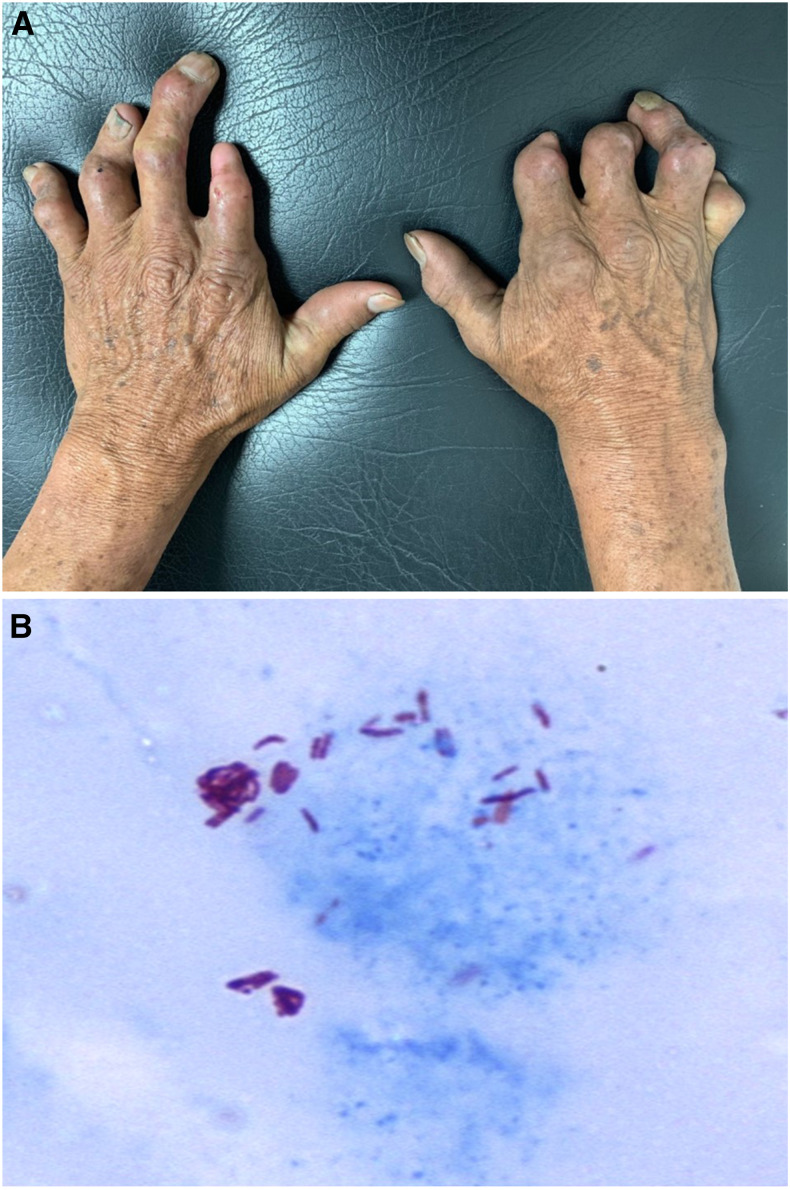
(**A**) Bilateral claw deformity of both hands, together with finger shortening and distal phalangeal reabsorption on several of them. (**B**) Skin slit–derived specimen directly applied to a slide and stained with Ziehl–Neelsen stain, showing multiple mycobacterial bacilli. This figure appears in color at www.ajtmh.org.

A 62-year-old hypertensive man, who lived his entire life in a remote rural area of Colombia (Caucasia, Antioquia) and used to hunt and eat Armadillos over several years when he was a child, presented to an outpatient clinic with a 13-year history of glove-like anesthesia and progressive deformity on both hands, associated with 5-kg weight loss, asthenia, adynamia, subjective fever, and osteoarticular pain. On physical examination, both hands showed a severe claw deformity (Figure 1A) with palm flattening due to severe thenar and hypothenar atrophy, together with finger shortening and distal phalangeal reabsorption on several of them. The sensory deficit compromised the palmar side of both hands but respected the territory of the radial nerve. Ulnar nerve thickening was evident on palpation of the right wrist, and Froment and bottle signs were positive on both hands. Skin examination revealed several post-traumatic scars, xerodermia, and diffuse skin thickening with multiple large, annular, hypopigmented, atrophic macules. Skin slit–derived specimens were obtained from elbows and earlobes according to local guidelines and were directly applied to a slide to be stained with Ziehl–Neelsen stain, showing multiple mycobacterial bacilli (Figure 1B). On the basis of clinical and bacteriologic findings, the condition was diagnosed as leprosy (Hansen’s disease) of the lepromatous type, and treatment with dapsone, clofazimine, and rifampin was started, following the WHO recommendations for multibacillary leprosy, presenting partial clinical improvement of the patient’s skin lesions and neuropathic symptoms, but not of the hand deformity.

Leprosy is a granulomatous disease caused by *Mycobacterium leprae* and is one of the major causes of preventable disability around the world. In the recent years, there has been an increase in the number of new leprosy patients with disability, specifically due to delay in diagnosis.^1^ People affected by leprosy often experience severe stigmatization because of its disabling consequences, and despite the availability of health facilities, there continue to be barriers toward leprosy diagnosis and early treatment that can prevent severe functional disabilities, especially on hands,^2^ and finally, the only way to improve function is reconstructive surgery.^3^
